# Supplementary material for: Dynamic Immune Response to Vibriosis in Pacific Oyster Crassostrea gigas Larvae during the Infection Process as Supported by Accurate Positioning of GFP-Tagged Vibrio Strains
Source: Microorganisms. 2021 Jul 17;9(7):1523. doi: 10.3390/microorganisms9071523 (PMC8303456; doi:10.3390/microorganisms9071523)

microorganisms

Dynamic Immune Response to Vibriosis in Pacific Oyster *Crassostrea gigas* Larvae during the Infection Process as Supported by Accurate Positioning of GFP-tagged *Vibrio* Strains

Dongdong Wang [1,*], Alfredo Loor [1], Lobke De Bels [2], Gilbert Van Stappen [1], Wim Van den Broeck [2] and Nancy Nevejan [1]

[1] Laboratory of Aquaculture & *Artemia* Reference Center, Department of Animal Sciences and Aquatic Ecology, Faculty of Bioscience Engineering, Ghent University, 9000 Ghent, Belgium
[2] Department of Morphology, Faculty of Veterinary Medicine, Ghent University, Salisburylaan 133, 9820 Merelbeke, Belgium

*** Correspondence:**

Dongdong Wang

Tel.: +32 476272489;

Fax: +32 476272489;

e-mail: dongdong.wang@ugent.be (D.D. Wang); dongdongwangqd@163.com

Figure S1. The stable toxicities of *V. splendidus* ME9-GFP (A) and *V. anguillarum* NB10-GFP (B) (10^6 CFU mL^{-1}) in experiment 1-4 on *C. gigas* larvae over 96 h exposure. Data are expressed as mean ± S.E (n=3). Values at each time point that are marked with a different letter are significantly different ($P < 0.05$, two-way repeated measures ANOVA).

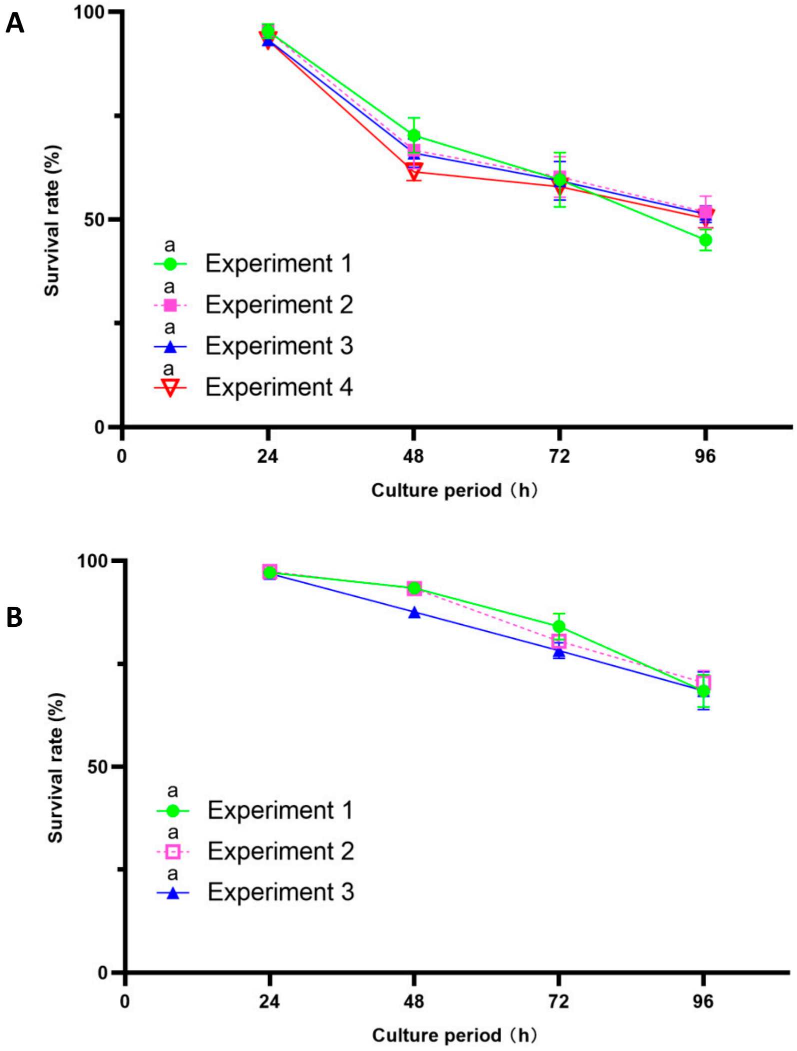

Supplement: Supplementary file 1 [file microorganisms-09-01523-s001.zip › microorganisms-1285983-supplementary.pdf]
